# Evaluation of a Physical Therapist-Delivered Technology Literacy Algorithm and Protocol for Older Adults: A Pilot Study

**DOI:** 10.7759/cureus.47865

**Published:** 2023-10-28

**Authors:** Christopher M Wilson, Sara K Arena, Lori Boright, Nicholas Duplissis, Michael Hilliker, John Krupa

**Affiliations:** 1 Physical Therapy, Oakland University, Rochester, USA; 2 Physical Medicine and Rehabilitation, Corewell Health, Southfield, USA

**Keywords:** evaluation for telehealth, physical medicine and rehabilitation, digital literacy, social determinants of health (sdoh), healthy aging

## Abstract

Introduction

Technology literacy is the ability to comfortably understand, use, and navigate digital devices. It is considered a “super social determinant of health”, and yet 39% of adults aged 65+ report not using a smartphone, and 25% of seniors still lack internet access. The purpose of this study was to examine the applicability of a physical therapist-delivered clinical assessment tool related to technological literacy and to identify relationships between technology utilization and perceptions related to sociodemographic factors in community-dwelling older adults.

Methods

A prospective mixed-methods observational-descriptive study where physical therapists (PTs) administered a newly designed technology literacy algorithm to older adults and evaluated the results of the algorithm. A convenience sample of 30 participants aged 65 and older was evaluated for their technology literacy. The exclusion criteria were if the person had a vision deficit, lived in a nursing home or extended care facility, was unable to fluently read and understand the English language, or was not willing to have an in-home visit by a licensed PT. After informed consent was obtained, the participant completed a Past Experience with Technology Questionnaire assessing participant confidence with technology usage and a demographic questionnaire. A PT data collector visited participants’ homes and administered a novel technology literacy algorithm. The PTs also provided subjective feedback after patient visits as to their perceptions of the algorithm. Inferential statistics were performed for key variables, including a Kruskal-Wallis test being utilized for variables with three or more levels and a two-sample Wilcoxon test being utilized for variables with two levels. The binary results were evaluated with chi-squared tests. Trends in distribution and measures of central tendency were analyzed for demographic data. Statistical significance was set at P<0.05 with a confidence interval of 95%.

Results

Participants (n=30) were evenly distributed with regard to age, and 66% of people had a college degree. Most were female, of the white race, and retired. There were statistically significant relationships between older age and decreased comfort level with using the internet (P=0.30) and sending messages (P=0.31), with individuals 80+ years old having a mean confidence of 6.78 out of 10. A statistically significant relationship was also found between higher income and increased confidence in browsing the internet (P = 0.07). Most qualitative data from physical therapist experiences included positive trends such as ease of use, efficiency, and confidence instilled. Constructive feedback included a lack of resources to assist with more advanced technology-related needs and recommendations to refine the algorithm when advanced needs were identified.

Conclusion

Technology literacy is a vital component of accessing health and medical care and maximizing the quality of that care, especially in the older adult population. The tools created may assist clinicians with identifying and addressing issues related to technology in older adults. This may help a patient navigate health issues that require the use of technology in their home. This study provided evidence that a PT-administered algorithm may be feasible to address technology literacy issues in the homes of older adults.

## Introduction

The rapid growth and expansion of technology have made technology literacy an essential function in daily life. With technology being used more frequently in healthcare than ever before, it is important for older adults to be able to possess the technological skills that will allow them to receive the best possible care from clinicians [1]. Technology literacy has been defined as the ability to comfortably understand, use, and navigate digital devices [2]. Inadequate technology literacy can lead to poorer self-management of health conditions. Some common issues with health technology utilization include difficulty navigating devices, a lack of stable internet connectivity, privacy concerns, self-efficacy, and cost [3]. There are many barriers that prevent older adults from interacting with technology, including cost, lack of knowledge, and privacy concerns [4]. In 2022, Feverio reported that 39% of adults aged 65+ did not use a smartphone, and 25% of seniors still lack internet access [5].

There are many benefits to utilizing technology in the healthcare setting, including cost, effectiveness, providing greater access to people in rural areas, and improving care for individuals with mobility limitations. Electronically conducted medical interventions have been shown to improve fall outcomes, decrease fall risk, and improve balance [6]. Research has shown that electronic health interventions can significantly improve quality of life and depression in individuals with health conditions such as cancer [7]. The use of technology to interact with individuals can also decrease the risk of spreading illnesses such as influenza or COVID-19 to high-risk populations, such as older adults. Telehealth visits have also been shown to save patients an average of $19-$121 per visit [8]. Technology literacy can increase access to care, promote the self-management of health conditions, and improve communication with loved ones [9].

Although there are many benefits to engaging with technology, there are many barriers that prevent some older adults from utilizing continued advancements in technology. These barriers include, but are not limited to, accessibility, reliability, cost, family support/assistance, and ease of learning [10]. With technology being used more often in healthcare now, it is important for older adults to be able to learn the technological skills that will allow clinicians to provide the best possible care for their patients. According to a 2021 study, the adoption rates of internet, smartphones, and broadband services decline significantly as an age group increases [11]. Streamlining the process of enabling older adults to problem-solve and navigate challenges that come with technology may make the process of implementing technology into the plan of care more efficient, which facilitates improved patient care.

Regarding the importance of the topic of technology usage, digital literacy and internet connectivity have been called “super social determinants of health” because they address all other social determinants of health [12]. Applications for housing, employment, and other assistance programs, all of which impact an individual's health, are often exclusively accessible online. The costs associated with equipping an individual to use technology for health promotion purposes are likely significantly lower than those associated with treating health conditions, and the benefits are believed to be long-lasting and impactful. Making efforts to improve technology skills and access are valuable tools to reduce disparities [13]. Clinical evaluation tools for assessing technology literacy may lead to increased adherence to home exercise programs, improved examination efficiency and better clinical outcomes, and adherence to a healthy lifestyle [14]. There is currently a need for better tools to assess technology literacy and barriers to technological utilization among older adults.

The purpose of this study was to examine the applicability of a physical therapist-delivered clinical assessment tool related to technological literacy and utilization and identify relationships between technology utilization and perceptions within the context of sociodemographic factors in community-dwelling older adults.

## Materials and methods

Research design

A prospective mixed-methods observational-descriptive study design was implemented where physical therapists administered a newly designed technology literacy algorithm and process to older adults and evaluated the results. This study was approved by the Oakland University Institutional Review Board (FY2021-377). Informed consent was obtained, and the rights of the participants were protected at all times during the study.

Study sampling criteria

Thirty individuals were recruited using a combination of recruitment flyers and reaching out to local community centers to invite adults over the age of 65 to participate in the study. Before enrollment, investigators screened all potential participants via phone to ensure each participant met the inclusion criteria and that no exclusion criteria were present. Inclusion criteria for participation in the study included being an adult age 65 or older, living in their own home or independent living, and having the willingness to have a licensed physical therapist (PT) come into the home to perform a technological assessment and provide recommendations. Exclusion criteria for this study included having impaired vision that limited their ability to read the required forms, living in a nursing home or extended care facility, unwillingness to have an in-home visit by a licensed PT, and an inability to fluently read and understand the English language.

Survey methods

The surveying of the participants included the use of a researcher-developed Past Experience with Technology Questionnaire (Appendix 1) before the home visit, as well as a basic demographic form. The basic demographic questionnaire collected information about the participants age, sex, marital status, annual income, and race. The Past Experience with Technology Questionnaire asked participants about their confidence with sending and receiving text messages (Likert scale), confidence with participating in virtual appointments (Likert scale), and whether they have access to someone in their home to help with tech issues (Range from Never to Always).

Research protocol

To identify trends in technology literacy among older adults, an algorithm was created to help identify potential barriers and offer resources to provide for participants during a one-time in-home visit conducted by a licensed PT.

Technology Literacy Algorithm

**Figure 1:**
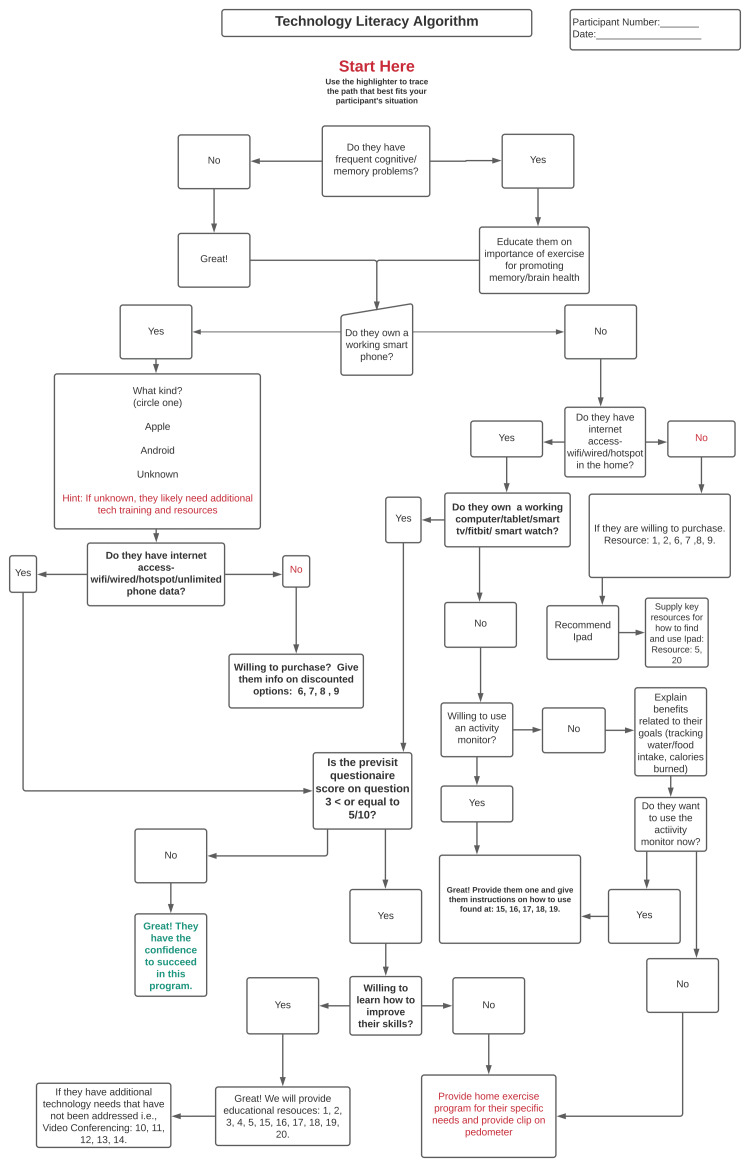
Technology Literacy Algorithm

**Figure 2:**
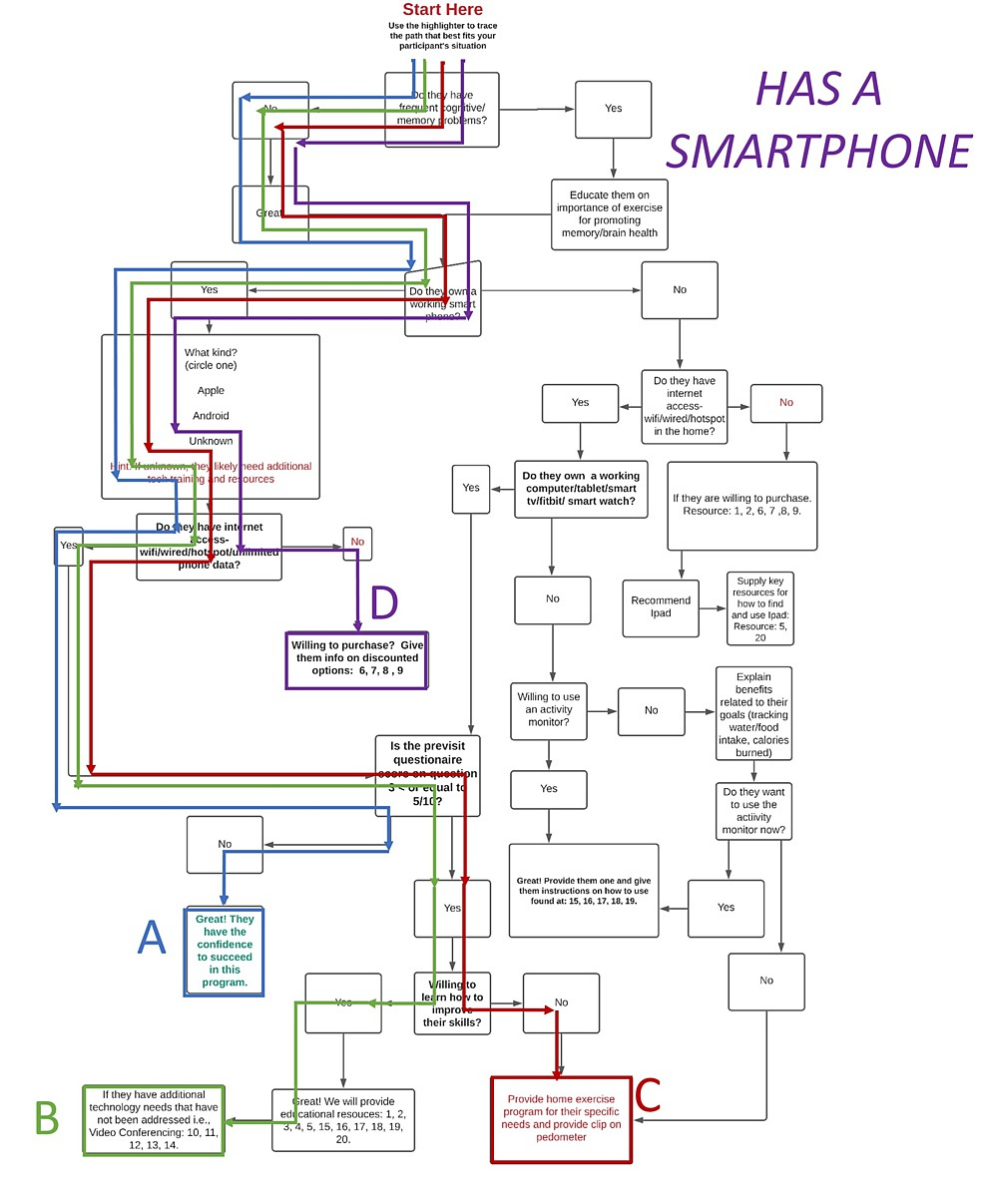
Possible Pathways When Participant Owns a Smartphone

**Figure 3:**
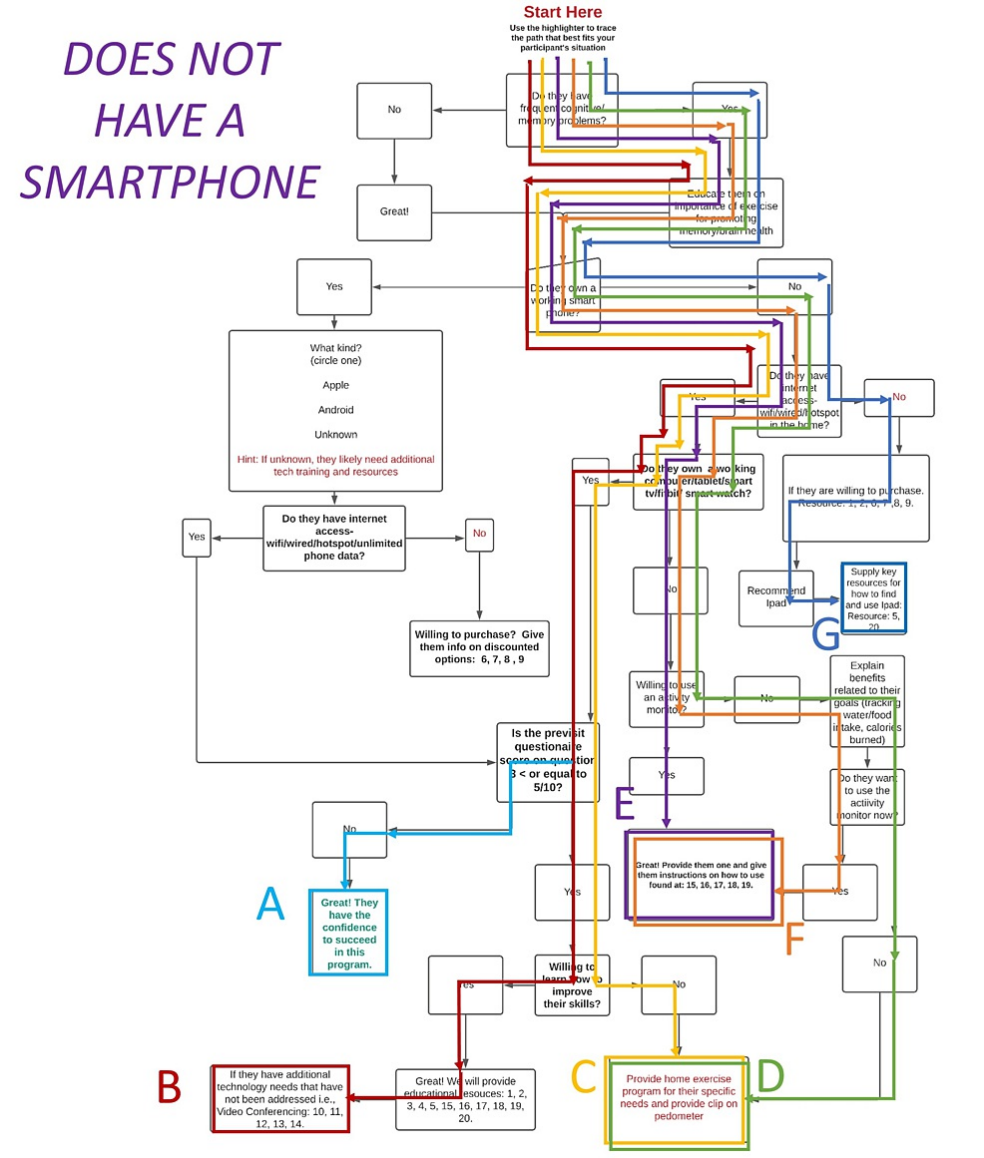
Possible Pathways When the Participant Does Not Own a Smartphone

The algorithm was designed purposefully to be quick and clinically applicable in the homes of older adults for administration by PTs to assess technology literacy (Figure 1). The algorithm is comparable to a flowchart in regard to providing the user with options of different paths to take to lead them to a predetermined group of resources intended to address the older adults’ technological issues in a timely and cost-effective manner. These pathways are designated in (Figure 2) the algorithm below for a participant that has a smartphone as: “A”, “B”, “C”, and “D” (Table 1). A participant that does not have a smartphone could progress through the algorithm (Figure 3) to pathway endpoints: “A”, “B”, “C”, “D”, “E”, “F”, or “G” (Table 2). As the therapist progressed down the algorithm, the participants were required to answer increasingly more specific questions related to the older adult’s technological skills or accessibility when managing aspects of technology such as a smartphone or reliable wireless internet. The data collectors were asked to trace the path on a paper copy of the algorithm during the session with the older adult participant. In addition, the PT data collectors were asked to record their subjective opinions about what they liked or disliked about using the algorithm at the conclusion of each session with participants.

**Table 1:** Decision Points and End Results if the Participant Owns a Smartphone

Path for “Has a smartphone”	Internet access?	Confidence browsing internet > 5/10?	Willing to learn to improve skills?	End result of pathway
A	Yes	No	N/A	No additional action needed
B	Yes	Yes	Yes	Provide educational resources
C	Yes	Yes	No	Provide home exercise program for their specific needs and provide clip on pedometer
D	No	N/A	N/A	If willing to purchase, give resources

**Table 2:** Decision Points and End Results if the Participant Does Not Own a Smartphone

Path for “No smartphone”	Internet access or wired hotspot?	Own a working computer/tablet/smart tv/Fitbit/smart watch?	Confidence browsing internet > 5/10	Willing to learn to improve their skills?	Willing to use an activity monitor?	End result of pathway
A	Yes	Yes	No	N/A	N/A	Sufficient confidence to succeed in the program
B	Yes	Yes	N/A	Yes	N/A	Provide educational resources
C	Yes	Yes	N/A	No	N/A	Provide home exercise program for their specific needs and provide clip on pedometer
D	Yes	No	N/A	N/A	No	Provide a home exercise program for their specific needs and provide a clip-on pedometer
E	Yes	No	N/A	N/A	Yes	Provide them with a wearable activity monitor and give instructions for use
F	Yes	No	N/A	N/A	Yes	Provide them with a wearable activity monitor and give instructions for use
G	No	No	N/A	Yes	N/A	If willing to purchase wireless internet access, provide resources for purchases and how to use an iPad

Training of data collectors

A virtual meeting was held to train the three PT data collectors in the performance of the technology literacy evaluation, proper utilization of the algorithm to assess the technological literacy of older adult participants, interpreting the past experience with technology questionnaire, and instructions on how to provide the resources listed at the endpoints of the algorithm to assist in addressing the deficits in technology literacy and resources among the participants. These data collector PTs were primarily trained by the principal investigator to ensure clarity and consistency throughout the course of data collection.

Data collection

Each data collection session was conducted by a licensed physical therapist during a one-time 90-minute visit to the participant’s home. Details of the visit flow, including data collection and the intervention, were as follows: Once the participant had expressed interest and verified eligibility based on inclusion/exclusion criteria, they were mailed two copies of the informed consent form, an investigator-generated eight-question demographic questionnaire, and an investigator-generated 10-question past experience with technology questionnaire regarding their comfort with and use of technology. During the in-home visit, the PT asked questions according to the algorithm and observed relevant technology (e.g., tablet, Wi-Fi router, computer, smartphone) within the participant’s home to discuss the different types of technology they currently use in their home, their proficiency with technology, and any issues they have had in the past related to technology usage. The PT marked the path on a paper copy of the algorithm based upon the participant's responses and technological resources. After this, the PT provided the participant with the resources and tools suggested by the respective paths taken in the algorithm (Appendix 2). The visit concluded with the PT answering any questions from the participant regarding the visit. Finally, the PT wrote down any perceptions or reflections related to the usefulness of the intervention. Upon completion of the in-person visit, the data was coded and entered for analysis.

Data analysis

Descriptive statistics were generated for demographic and outcome variables. Statistical analyses were performed by a statistician using SAS version 9.4 software for Windows. A Kruskal-Wallis test was utilized for variables with three or more levels, and a two-sample Wilcoxon test was utilized for variables with two levels. The binary results were evaluated with chi-squared tests. When the variances of the groups were not equal (only for those with vision and hearing problems), a Fligner-Policello version of the Wilcoxon test was utilized. Finally, if the cell size in the 2x2 table was less than 5, a Fisher’s exact test was used. Statistical significance was set at p≤0.05 with a confidence interval of 95%. Demographic data was analyzed descriptively using demographics and survey responses, percentages, frequency counts, and measures of central tendency. Frequency counts were obtained for each completed technology literacy algorithm to identify how often each endpoint of the algorithm was reached. Qualitative analysis was performed using the constant comparative method regarding data collector feedback and experiences to determine trends in their responses.

## Results

Demographic Data

Thirty participants took part in this study. See Table 3 for demographic results.

**Table 3:** Demographic Data GED: General Educational Development high school equivalency test

Category	Total (n=30)	% of Total
Age		
65-69	8	26.7%
70-74	7	23.3%
75-79	6	20%
80+	9	30%
Sex		
Male	10	33.3%
Female	20	66.7%
Race		
Black/African American	4	13.3%
White/Caucasian	25	83.3%
Mixed	1	3.3%
Marital Status		
Married	20	66.7%
Single – Previously Married	6	20%
Widowed	4	13.3%
Highest Level of Education		
High School/GED	2	6.7%
Some College	5	16.7%
Associate Degree	3	10%
Bachelor Degree	12	40%
Master Degree	8	26.7%
Employment Status		
Full Time Employed	2	6.7%
Retired	28	93.3%
Annual Income		
10-25K	2	6.7%
25-50K	5	16.7%
50-75K	4	13.3%
75-100K	6	20%
100-150K	3	10%
Prefer Not to Answer	10	33.3%

Participants provided responses to questions related to their use of technology and their memory and concentration (Table 4).

**Table 4:** Responses to Various Technology Topics

Item	Frequency count of responses				
I have someone I can use to help with tech issues	Always=9	Almost always=7	Sometimes=12	Almost never=0	Never=1
What do you use to browse internet	Wi-Fi=28	Wired/dial up=0	Hotspot=0	Phone data=4	Unknown=2
How do you learn new technology (Select all that apply)	Family=26	Friends=12	Yourself=20	Professional=6	Internet=16
Difficulty remembering or concentrating well	Never=1	Rarely=5	Sometimes=9	Often=0	Always=0

A statistically significant relationship was identified when comparing the relationship between age category and comfort with the internet (P=0.030) and sending texts (P=0.031) with individuals 80+ years old, with a mean confidence of 6.78 out of 10 (Table 5). No statistically significant relationship was found between age and having a source to help with technology or with participants’ motivation or confidence with technology (P>0.05). There was a statistically significant relationship between sex and confidence using telehealth (P=0.039), with men reporting a mean confidence of 6.75 out of 10 and women reporting 8.75; no other relationships examining sex and other variables were statistically significant.

**Table 5:** Participant Responses on Confidence and Comfort *only participants who used telehealth in the past; bold denotes statistical significance at P<0.05

Mean (SD)	N	All participants	Age					Sex			Race			Marital Status		
			65-69	70-74	75-79	80+	P-value	Male	Female	P-value	White	Non-White	P-value	Not Married	Married	P-value
Comfort reading and sending text messages (1=least, 10=most)	30	8.5 (2.86)	9.19 (0.75)	8.57 (3.36)	7.33 (2.16)	6.00 (3.39)	0.031	7.90 (2.33)	7.63 (3.14)	0.666	8.20 (3.49)	7.62 (2.79)	0.254	7.65 (2.73)	7.75 (2.99)	0.822
Confidence browsing the internet (1=least, 10=most)	30	8.12 (2.13)	8.81 (1.31)	9.57 (0.77)	7.50 (2.07)	6.78 (2.68)	0.030	8.60 (1.07)	7.88 (2.49)	0.685	8.40 (3.05)	8.06 (1.98)	0.314	8.05 (2.14)	8.15 (2.18)	0.750
Motivated to learn new technology (1=least, 10=most)	27	7.91 (1.43)	8.64 (1.60)	7.50 (1.05)	7.50 (0.84)	7.88 (1.81)	0.278	7.88 (1.46)	7.92 (1.46)	0.624	8.00 (1.22)	7.89 (1.50)	0.873	8.65 (0.82)	7.47 (1.55)	0.027
Confidence participating in telehealth (1=least, 10=most)	19*	7.73 (2.28)	8.40 (2.07)	8.40 (1.67)	6.67 (3.21)	9.33 (0.58)	0.503	6.75 (2.50)	8.75 (1.66)	0.039	8.00 (1.63)	8.33 (2.19)	0.453	9.25 (0.96)	7.92 (2.19)	0.261

When considering self-reported race, three out of 25 (12%) individuals who were White/Caucasian were less likely to utilize professional assistance than those of races other than white (three out of 25 (60%)) (P=0.041); however, the small sample size of the non-white individuals should be noted. No other statistically significant relationships were examined when considering race.

A statistically significant relationship was noted between marital status and motivation to learn new technology (P=0.027), as those who were not married reported a mean confidence of 8.65 out of 10, and those who were married reported a mean confidence of 7.47.

As it relates to education, there was no statistically significant difference between those with a higher degree of education and those without as it relates to using the internet, sending texts, having help with technology, being motivated to use new technology, or confidence in using telehealth (P>0.05).

Four people reported unresolved vision or hearing problems. Those with vision or hearing issues were more likely to have a convenient resource for help with technology (Likert scale: 1 = always, 5 = never) as compared to those without vision or hearing problems (mean score: 1.25 vs. 2.44, P=0.001).

A statistically significant relationship was found between income and confidence in browsing the internet (P=0.07), with those with lower income generally reporting less confidence than those with higher income. Similarly, those with lower incomes were significantly less confident in sending and reading text messages than those with higher incomes (P=0.01).

As it relates to income, those with lower incomes were significantly less likely to have a convenient resource for help with technology as compared to those with higher incomes (P=0.045). No other statistically significant relationships were identified as relating to income.

A statistically significant correlation was found between having a convenient resource for help with technology and confidence in using the internet (P=0.045), with those who had nobody to help with technology being less confident in their ability to use their device to browse the internet (Table 6). Additionally, there was a statistically significant correlation between confidence in using telehealth and motivation to learn new technology (P=0.006). 

**Table 6:** Spearman Correlation Bold denotes statistical significance at P<0.05

	Variable = Correlation Coefficient (P-value)		
Have someone to help with technology	Comfort reading and sending text messages = -0.24 (P=0.203)	Confidence browsing the internet = -0.37 (P=0.045)	Motivated to learn new technology = -0.08 (P=0.684)
Confident with telehealth	Have someone to help with technology = -0.21 (P=0.434)	Confidence browsing the internet = 0.29 (P=0.279)	Motivated to learn new technology = 0.68 (P=0.006)

A statistically significant relationship was found between previous use of telehealth and confidence in using the internet (P=0.039), with those who had used telehealth before being more confident in using the internet than those who had not used any form of telehealth before.

Algorithm results

Of the 30 participants who took part in the study, 28 reported owning a smartphone. Of these 28, 26 participants went along path “B”, which shows that they had access to the internet and were confident in using it. Two participants went along path “C”, which shows that they had access to the internet but were not confident in using it, but they were willing to learn.

Of the two participants who reported not owning a smartphone, both went along path “C” on this side of the algorithm, which shows that they reported having access to the internet, owned a working tablet or personal computer, were not confident in using it, but were willing to learn.

Qualitative comments and trends

The PT data collectors provided comments that detailed the positive and negative aspects of using the algorithm with the participants.

Positive trends included the algorithm’s ease of use, improvements in participants’ perceived self-efficacy with technology usage, and successful identification of someone with technological needs. Some data collectors reported that the algorithm helped instill confidence in the participants, who previously expressed concerns about not being well prepared to utilize basic technology.

Negative responses included that the resources occasionally were not helpful with specific technology-related issues experienced by the participants, and the algorithm was not able to identify some more advanced or complex technology needs of the participants. Some data collectors suggested expanding the algorithm in order to more accurately assess the more specific needs of the participants.

## Discussion

The purpose of this study was to examine the applicability of a physical therapist-delivered algorithm related to technological literacy and to identify relationships between technology utilization and perceptions related to sociodemographic factors in community-dwelling older adults. Our results determined that the tools created in this study may assist clinicians in identifying and addressing their patients’ opportunities related to technology and telehealth; this may result in future improved health outcomes and cost savings. Written responses from the data collectors showed both positive and negative aspects of the algorithm, and a majority of the data from the results support previous research in regard to sociodemographic influences on technology utilization [15,16].

The results of this study show that females are more likely to have confidence using telehealth than males. According to a study by Escoffery in 2017, females are more likely to seek out health information online and to have a mobile app for health [15]. This is consistent with our findings that females are more confident with technology than males. Women may be more health-conscious than men and, therefore, may be more likely to use a health app related to diet and exercise [17]. This could result in females having an increased familiarity with technology with regard to health compared to males.

A study by Harris and colleagues found that being widowed resulted in significantly lower technology literacy skills [16]. This goes against the findings from our study, which found that unmarried individuals reported significantly higher mean confidence for learning new technology than married individuals. One reason unmarried individuals have less technology literacy skills could be that they do not have a spouse to rely on for help with technology issues. On the other hand, a possible explanation for unmarried individuals having more technology literacy skills could be that since they do not have anyone to rely on for help, they must figure out how to navigate technological challenges themselves, resulting in more experience with technology than they would have otherwise.

Results from this study showed that individuals with lower incomes were significantly less confident with technology utilization than those with higher incomes. This information can be used to better understand the technological difficulties that individuals with lower incomes might face. Providing helpful resources to this population may result in more efficient use of technology.

Results from this study showed that individuals with nobody to help them with technology issues were significantly less confident using their device to browse the internet. This information can be used to encourage clients to find a family member or close friend to help them with technology issues, which may result in the individual having more confidence with internet-based medical issues, such as telemedicine and viewing online medical records.

Study limitations

The sample size of participants is relatively small (n=30), which can make it difficult to generalize this information to a larger population. Within this study, there was no assessment of a correlation between health outcomes and technological literacy. Test-retest reliability as well as comparative validity testing were not available for this study. The geographic location of the participants being limited to southeastern Michigan reduces the generalizability of the results, as location may impact access to and competency with technology. Within the demographic questionnaire, the marital option of previously married could have been construed as widowed or divorced or may have been otherwise open to interpretation. Furthermore, this document also asked for the participant’s “sex” without including “gender” as a category. This could have skewed the information to not be inclusive of differing gender identities.

Future research 

The resources provided to the PTs based on the results of the technology assessment could be examined for their effectiveness in advancing technology literacy after the algorithm is administered, as well as generalized for participants outside the Southeast Michigan region. Also, an expanded version of the algorithm to include anticipated future advancements in technology could be created as more of a “maintenance” assessment of literacy to keep knowledge updated after the initial assessment was administered. In addition, it would be beneficial to assess the algorithm’s direct applicability to physical therapist practice.

## Conclusions

Technology literacy is a vital component of adapting to advancements in medical care and maximizing the quality of that care, especially in the older adult population. The tools developed from this study may eventually be used to streamline the evaluation of technology literacy skills in older adults. By having the knowledge and tools necessary to use technology for health-related purposes, older adults may be able to navigate and access health-related interactions. This study serves as the framework for allowing any older adult who may have limited knowledge of technology usage to employ new strategies to enhance their care.

## Appendices

Appendix 1

**Table 7:** Past Experience With Technology Questionnaire

This document is intended to assess your current and/or previous experience with technology usage. Please circle your responses below. You can skip any questions that you don’t feel comfortable answering.	
1. Do you own a smartphone?	Yes. No.
If yes, is it an Apple, Android, or unknown? (Circle option)	Apple. Android. Unknown.
2. Circle the number that best represents how comfortable you are with your ability to send and read your text messages. (1=Extremely Low Confidence;10=Extremely High Confidence)	1 2 3 4 5 6 7 8 9 10
3. Circle the number that best represents how confident you are in your ability to use your phone/computer/tablet to browse the internet, YouTube, Facebook, etc. (1=Extremely Low Confidence;10=Extremely High Confidence)	1 2 3 4 5 6 7 8 9 10
4. Which of the following do you use to browse the internet in the home? Circle all that apply.	Wi-Fi. Wired connection/ethernet/dial-up. Hotspot. Phone data. Unknown.
5. If training was provided to you, would you be willing to use a wearable activity monitor? (such as a Fitbit)	Yes. Maybe. No.
6. How do you usually learn how to use new technology- help from family members, friends, figure it out yourself, assistance from some professional? Circle all that apply.	Family. Friends. Yourself. Professional. Internet. Other: _________
7. Do you have any unaddressed vision or hearing problems that make it difficult for you to utilize technology such as a phone, computer, tablet, activity monitor?	No. Yes, I have vision issues. Yes, I have hearing issues. Yes, I have issues with both vision and hearing. If issues you have are corrected, please specify: _________________________________
8. Circle the number that best represents how motivated you are to learn how to use new technology. (1=Not at all motivated;10=Extremely motivated)	1 2 3 4 5 6 7 8 9 10
9. How often do you have difficulty remembering or concentrating well? (Circle one response)	Never Rarely Sometimes Often Always
10. Have you ever had a telehealth visit over the phone, computer, tablet?	Yes No
If yes, how confident are you in your ability to participate in these visits? (Circle one response) (1=Extremely Low Confidence;10=Extremely High Confidence)	1 2 3 4 5 6 7 8 9 10
If you have not participated in a telehealth visit before, would you be willing to try it? (Circle one response)	Yes. Maybe. No. Unsure.

Appendix 2

**Table 8:** Resource List Provided to Participants Based on Results of Algorithm Numbered items in this appendix correspond to resources to be provided at the end of each pathway on algorithm.

Resources to be Provided Based Upon Results of Algorithm	
Topic	Web Link
Setting up wireless internet in the home	
1. Video	https://www.youtube.com/watch?v=UBqy9ZIxa_Y
2. Written Instructions	https://edu.gcfglobal.org/en/basic-computer-skills/how-to-set-up-a-wifi-network/1/
Pairing a Bluetooth device	
3. Pairing an Android device	https://support.google.com/android/answer/9075925?hl=en#zippy=%2Coption-use-the-settings-app-all-bluetooth-accessories
4. Video on how to turn on Bluetooth on phone or tablet	https://youtu.be/W3WcPz5f3Ss
5. Pairing an Apple device	https://support.apple.com/en-us/HT204091
Discounted phone and internet options	
6. Informational article	https://www.aarp.org/home-family/personal-technology/info-2021/programs-older-adults-computers-internet-access.html
7. Xfinity discount program	https://www.xfinity.com/learn/internet-service/ebb#eligibility
8. American Association of Retired Persons (AARP) discounted phone options	https://www.aarp.org/benefits-discounts/all/consumer-cellular-10002/
9. TracFone support and information	https://www.tracfone.com/support
Video Conferencing	
10. Skype setup video	https://www.youtube.com/watch?v=Bhwens7f_tg
11. Written instructions on how to make a Skype call	https://support.skype.com/en/faq/FA10613/how-do-i-make-a-call-in-skype
12. Informational article on Skype calling	https://www.businessinsider.com/how-to-set-up-a-skype-conference-call
13. Scheduling a Skype meeting	https://www.youtube.com/watch?v=k_uesMV78eE
14. How to use Zoom video written instructions	https://support.zoom.us/hc/en-us/articles/206618765-Zoom-video-tutorials
Activity Monitor	
15. Troubleshooting a Garmin Activity Monitor	https://support.garmin.com/en-US/?faq=TVtJ74jg5E2yJzM3kS8iA9
16. Video on how to download the Garmin Connect app on Android or iPhone	https://youtu.be/-5gV4FG65nA
17. Video on how to create a Garmin account and connect watch to app	https://youtu.be/KZypveM4RFM
18. Garmin support center for Garmin Vivofit 4 with written instructions	https://support.garmin.com/en-US/?productID=582444&tab=topics
19. Garmin VivoFit 4 owner's manual	https://www8.garmin.com/manuals/webhelp/vivofit4/EN-US/vivofit_4_OM_EN-US.pdf
iPad resources	
20. iPad user guide	https://support.apple.com/guide/ipad/welcome/ipados
